# Statistical downscaling reproduces high-resolution ocean transport for particle tracking in the Bering Sea

**DOI:** 10.1038/s41598-026-37904-1

**Published:** 2026-02-04

**Authors:** Trond Kristiansen, Jordan Miller, Momme Butenschön

**Affiliations:** 1 Farallon Institute, Petaluma, CA USA; 2 Actea Inc, San Francisco, CA USA; 3 CMCC Foundation - Euro-Mediterranean Center on Climate Change, Bologna, Italy

**Keywords:** Statistical downscaling, Lagrangian particle tracking, Bering Sea ocean currents, Mesoscale ocean dynamics, Ocean reanalysis, Climate sciences, Ecology, Ecology, Ocean sciences

## Abstract

Understanding ocean transport is critical for applications ranging from fisheries to chemical plume tracking and carbon dioxide removal modeling. However, available hydrodynamic data often lack the spatial resolution needed for effective transport simulations. We apply statistical downscaling to coarse-resolution ocean reanalysis and atmospheric wind data, reconstructing fine-scale fields validated against high-resolution dynamic models in the Bering Sea. This enables the prediction of transport patterns without the need to run high resolution physics simulations, saving computational costs and time. We examined five years of high-resolution, statistically downscaled ocean currents and surface winds and found that the correlation of ocean current and wind components with GLORYS and ERA5 reanalysis models were r = 0.87 and r = 0.98. The Liu-mean skill score was 0.75 for ocean current velocity. Okubo–Weiss analyses showed comparable vorticity and shear between downscaled and dynamical models. The Finite-time Layupanov Exponent analysis showed consistent Lagrangian Cohesive Structures across datasets. Multi-year particle tracking using both downscaled and reanalysis forcing showed consistent relative separation distances with mean Bhattacharyya coefficient of 0.720 ± 0.133. The demonstrated parity in dispersal patterns indicates statistically downscaled approaches can substitute dynamical models for large-scale applications. Future work should validate these results across diverse oceanographic regimes and incorporate biogeochemical feedback mechanisms.

**Supplementary Information:**

The online version contains supplementary material available at 10.1038/s41598-026-37904-1.

## Introduction

Understanding and predicting the movement of substances in the ocean is a critical component of oceanographic research and environmental management. Lagrangian particle tracking has become a fundamental technique for studying ocean transport processes, with applications ranging from oil spill prediction^1^ to understanding marine connectivity patterns^1^ to tracking dispersal of contaminants such as plastics^2^ or locating a person lost at sea^3^. These applications require models that provide both accuracy and computational efficiency such as the ocean reanalysis GLORYS^4^ and atmospheric reanalysis ERA5^5^. Such dynamic reanalysis offers high-resolution, physically consistent representations of ocean currents and atmospheric conditions, but are typically restricted to specific historical periods. There are many cases where the only hydrodynamic fields available for the period and area of interest are too coarse to model the transport of interest. For example, often only coarse global models are available for either the historical periods or for future climate projections including outputs from the Coupled Model Intercomparison Project (CMIP^6^) where the resolution in the ocean is around 1 by 1 degree longitude and latitude. In contrast, realistic Lagrangian studies that quantify how e.g. chemical dispersal is affected by changes in ocean currents under different climate scenarios, require high-resolution (typically ≤ 10 km resolution) climate projections capable of resolving key ocean transport features such as mesoscale eddies and associated vertical velocities, which are fundamental drivers of transport and dispersion, that cannot be accurately captured at coarser resolutions^7^. Dynamic models such as the Regional Ocean Modeling System (ROMS^8^) or the Nucleus for European Modelling of the Ocean (NEMO^9,10^) are perfectly capable of downscaling (to increase the resolution) historical periods or climate projections, but their significant computational demands restrict their applications to limited geographic regions. For climate downscaling, the computational cost of using dynamic models also limits the number of climate models and scenarios to consider. This is particularly challenging when modeling broad transport processes or conducting ensemble analyses that require repeated model runs across vast spatial and temporal domains as in the monitoring, reporting and verification (MRV) of marine Carbon Dioxide Removal (mCDR^11^). These applications require high-resolution, meso-scale resolving, physical–chemical information to quantify the level of CO_2_ removed from the air-sea interface and sequestered at depth for decades into the future^12^. Understanding the long-term sequestration potential and impacts on the marine ecosystem necessitates modeling interventions decades into the future across various climate scenarios, while also resolving fine-scale physics. Applications like mCDR are geographically unrestricted and therefore a considerable challenge for dynamical approaches.

This study introduces an alternative solution to dynamic modeling for marine spatial dispersal analysis that employs a statistical approach, facilitating a reduction in complexity of model setup and computational cost, while ensuring a realistic solution. Recently, a few studies^13,14^ have applied statistical downscaling of vector fields to atmospheric wind using topography as a co-variate, however downscaling of ocean current fields has to our knowledge not been previously published. Statistical downscaling of climate projections at coarser resolution can be performed using existing historical dynamic high-resolution reanalysis or hindcasts as the statistical baseline^15,16^. The scalability and speed of statistical modeling offer opportunities to explore the efficiency, and potential ecosystem impacts from e.g., mCDR interventions, future oil spills, pollution, fate of chemical compounds etc. under multiple climate scenarios and to quantify model and scenario uncertainties. Here, we validate our assumption that a statistical model for ocean currents and atmospheric wind can efficiently and realistically generate the correct physical features of a dynamic model. We discuss model assumptions and compare the results between dynamic and statistical model outputs. Assuming this approach effectively reproduces the dynamic model results, it would enable the reuse of a single dynamic model’s vector outputs of ocean currents and wind to downscale multiple climate models and scenarios for the future. When combined with CMIP6 model outputs, this method offers an efficient and realistic approach to providing detailed climate information for Lagrangian studies including coastal domains globally.

This study (1) presents the methods used to downscale and evaluate the results, (2) describes the methods used to simulate drift and dispersal of water parcels using both downscaled and original reanalysis outputs, and methods for comparison, and (3) discusses the applicability of using downscaled products for dispersal tracking.

## Methods

### Methods overview

**Fig. 1:**
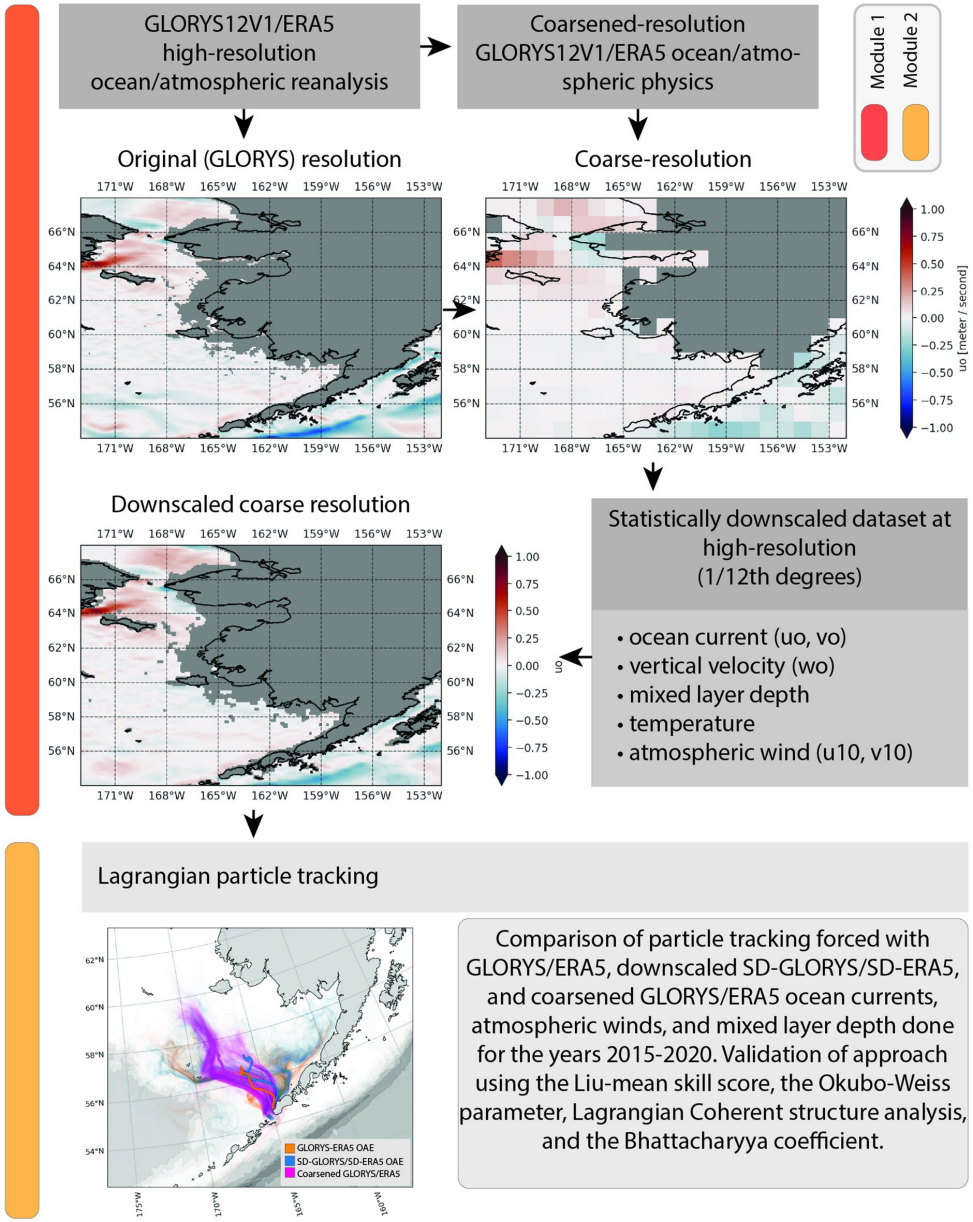
Visualization of the model system presented in this study. The two reanalyzes, GLORYS for the ocean^12^ and ERA5 for atmosphere, represents the dynamic models. These input data were made coarse to represent the typical resolution of ocean (1° × 1°) and atmosphere climate data (3° × 3°) and then used as input to the downscaling methodology. The resulting downscaled ocean currents at multiple depth levels, surface wind, and mixed layer depth were used to track the concentration of hypothetical ocean alkalinity enhancement solution released along the coast of Aleutians East Borough for the years 2015–2020. The same simulations were performed using the original GLORYS and ERA5 as well as coarsened GLORYS and ERA5 as input and the results were compared to quantify the skill of the resulting spatial–temporal distribution for each year. Figure created with Python v3.11.9 using modules Cartopy v0.23.0, Seaborn v0.13.2, and Matplotlib v3.9.2.

The modeling approach outlined in this study necessitates the use of existing dynamic atmospheric and ocean models as training data, such as the global ocean reanalysis GLORYS^4^ and the atmospheric equivalent ERA5^5^. These models provide historical high-resolution data that serve as the statistical baseline for the training of the bias correction and statistical downscaling methodology^16^. The downscaling transforms coarser resolution data, including climate model outputs to high-resolution, which can then be used as forcing for Lagrangian particle tracking applications (Fig. 1).

This study focuses on validating how well downscaled ocean currents and atmospheric wind fields realistically represent the dynamic physical characteristics of oceanic and atmospheric variability and reproduce their key physical features. The ability to reliably reproduce high-resolution current patterns from coarser inputs is essential for our capacity to provide efficient high-resolution current and wind fields. Therefore, it is essential that particle tracking performed using outputs from a statistical model and a dynamical one remain consistent. Here we validate the statistical downscaling component by using a known output as input to have a “true” solution for the purpose of validation. Specifically, we coarsened (reduced the resolution of) the GLORYS^4^ dataset for the years 2015–2020 to 1 × 1 degree longitude-latitude. We then used the coarsened dataset as input to the downscaling methodology to reproduce the high-resolution (1/12th degree longitude-latitude) ocean currents, before comparing the output with the original.

The methodology and model setup are represented as two modules (Fig. 1): Module (1) Bias-correction and statistical downscaling of coarse resolution model data (e.g., atmospheric and ocean reanalysis) to high-resolution, and Module (2) Particle tracking module.

#### Module 1

*Bias-correction* Global climate model (GCM) and Earth System Models (ESMs) outputs from CMIP6 are typically presented on various native grids and grid resolutions, ranging from 50 to 300 km. Ocean components are often provided at resolutions of 1° × 1° (longitude and latitude), while the atmosphere is represented at 2.5°-3.0° × 2.5°-3.0°. Global hindcast models, such as the Ocean Reanalysis System 5 (ORAS5) and the CMCC C-GLORS^17^, often have resolutions of ¼ degree. To use such coarse resolution projections for understanding historical or future aspects of ocean dispersal, it is essential to enhance their resolution to realistically represent the coastal meso-scale dynamics and variability of the flow fields. This is done by first bias-correcting the inherent differences between the coarse resolution model and the high-resolution historical reference data at the grid of the coarse model. In this study, we used the ocean reanalysis GLORYS at 1/12° resolution to assess biases and to represent the historical ´observations´, referred to as the reference data. The GLORYS reanalysis incorporates historical data (such as satellite, CTD, XBT, and buoys) from 1993-01-01 to 2019–12-31 and is a state-of-the-art hydrodynamic model provided as an operational service by the Copernicus Marine Service (marine.copernicus.eu). The bias correction was performed for the historical period 1993–2015 using detrended quantile mapping (DQM^18,19^). Multiple bias-correction methods such as the Equilibrium Quantile Mapping^20^ and Delta Quantile Mapping^21^ exists of varying complexity^22^, but the DQM represents one of the most commonly used approaches that preserves the mean climate change signal from original simulations, avoids extrapolation issues by working on historical ranges, maintains overall projected changes while correcting biases, and are computationally efficient^22^. The DQM method eliminates biases across all quantiles, aligning the model data distribution with the reference data, which ensures that the variability and range of values conform to the reference data. This alignment is achieved by creating a transfer function that minimizes the difference between the cumulative density function of the observed and modeled detrended data. Standard quantile mapping fails to preserve trends in climate projections because it overlooks the time dependency of distributions and applies a fixed transfer function to data whose mean changes over time. Detrended quantile mapping addresses this issue by first separating the climate signal into normalized and residual components, applying bias correction only to the normalized variations while preserving the original long-term changes and year-to-year fluctuations in the residual component, which is then added back after correction^23^. Since there will be no bias when using coarsened GLORYS data, and we need to validate the bias correction approach separately, we bias-corrected the CMCC-CM2-SR5 CMIP6 model for the training period (1993–2015) and for the validation period (2015–2020). We then compared the original CMCC model, the bias corrected CMCC model, and the GLORYS reanalysis for the same periods (Supplementary Figs. 1, 2, 3 and 4) to show that the bias correction consistently improves the output for the training and the validation period.

*Statistical downscaling (SD)* applies the DQM algorithm to establish a statistical relationship between the historical high-resolution ocean reference data and the bias-corrected model data. When applied to climate models, this method transfers coarse resolution bias-corrected model data to fine-scale spatial patterns, while preserving the bias-corrected climate change signal and extreme value trends from the original coarse model projections^15,24,25^. As with the bias-correction methodology, the training of the SD uses reference model values from 1993–2015 while the downscaling is performed for the period 2015–2020. The SD uses the GLORYS ocean reanalysis as reference to capture the sub-grid variability when transforming bias-corrected coarse resolution model projections (e.g., ESMs for the past and into the future) to high-resolution. The highest resolution we can downscale to is not limited by the methodology itself but primarily constrained by the resolution of the reference data (GLORYS, ERA5). The downscaling of ocean currents (*uo*, *vo*) was executed at fixed depth levels (5, 10, 20, 30, 50, 75, 100, 150, 200, 500, and 1000 m). Downscaling was also executed for ocean currents along the seafloor where each grid point had a unique depth level (see^16^ for more details). The downscaling of mixed layer depth (MLD) was done for the single depth varying field it represents.

#### Module 2

Lagrangian particle tracking was used to quantify the time-dependent dispersal of neutrally buoyant water parcels as these were transported with the ocean currents in a stochastic environment. The Lagrangian concept, contrary to the Eulerian perspective that observes a fixed point, tracks the position of a water parcel as it moves through space and time. The velocity of a water parcel at a known initial point can be interpolated from the flow field that contains it, and the trajectory can be found by numerical integration of small, discrete timesteps where the location is updated based on the calculated velocity at each timestep^26^. Particle tracking was implemented using the well-tested Python framework, Opendrift^26^. In addition to the advective flow, particles are mixed vertically in the surface layer by wind-stress induced turbulence at the air-sea interface using a time-step of 1800 s for advection and 300 s for vertical mixing. Vertical mixing processes require significantly smaller timesteps than advection due to numerical stability constraints and the well-mixed condition requirement^27^. Vertical mixing from wind-driven turbulence was confined to the MLD assuming stratification regimes. The wind-driven mixing was calculated following the K-Profile Parameterization (KPP)^28^ that unifies various mixing processes in both the ocean surface boundary layer and interior. Here, the turbulent diffusivity (*K*) above a surface boundary layer depth (MLD) is expressed as the product of length scale (MLD), turbulent velocity scale *w* and a non-dimensional shape function *G*:

1 $$K = MLDw\left( \sigma \right)G\left( \sigma \right)$$

Here, we used the parameterization for *K* as implemented in Opendrift^26^ where we assume stable/neutral stratification conditions. We also add stochastic horizontal and vertical diffusion (random kicks) using fixed background diffusivity values (horizontal 1 ms^−2^ and vertical 1e-5 ms^−2^)^29^ multiplied by a random number between 0–1. This creates stochastic spatial differences between particles released at the same location.

Particles in the surface layer (upper 30 m) are exposed to Stokes drift or the net displacement of water parcels caused by surface wind-driven wave motions, which can be important for vertical mixing in the upper ocean^30^. Wave induced propagation of water parcels move in circular, not horizontal, orbits in the direction of the wave. Here, we use a monochromatic single-wave spectrum and the surface wind speed and direction to parameterize Stokes drift speed and direction^31^. The advection of particles was forced with statistically downscaled horizontal surface wind (SD-ERA) at daily temporal resolution and downscaled monthly ocean currents at multiple depths (Fig. 1). To create a three-dimensional water column, the depth levels were stacked prior to input to OpenDrift. The MLD was independently downscaled, whereas the vertical velocity was calculated based on the principle of mass conservation, expressed through the continuity equation. For an incompressible fluid such as seawater, this relates vertical velocity to horizontal divergence and vertical velocity can be expressed as:

2 $$\frac{\partial w}{{\partial z}} = - \nabla \cdot V = - \left( {\frac{\partial u}{{\partial x}} + \frac{\partial v}{{\partial y}}} \right)$$

where $\nabla \cdot V$ is the horizontal divergence and u and v are the eastward and northward velocity components, and w is the vertical velocity (positive upward). Bottom boundary ($z=-h$):$w=-V\cdot \nabla h$ and surface boundary ($z=\eta$): $w=\frac{\partial \eta }{\partial t}+V\cdot \nabla \eta$. Here, *h* is bottom depth and $\eta$ is the free surface.

### Particle tracking simulations

The Bering Sea serves as our modeling site characterized by a vast, shallow continental shelf in the east and north, deep basins exceeding 4,000 m in the southwest, strong currents, seasonal ice cover, pronounced climate variability, and dynamic physical interactions between ocean and atmosphere. For these waters we downscaled the meridional and zonal ocean currents (SD-GLORYS) at several discrete depth levels with a monthly temporal resolution for the period 2015–2020. Atmospheric surface wind was downscaled at a daily temporal resolution (SD-ERA5), offering the frequency needed to simulate wind-driven turbulent mixing of the water column and surface Stokes drift. For the ocean, the underlying dynamic model for GLORYS calculates the physics at temporal resolution of minutes but the outputs are provided as monthly averages. We use these data as input to the downscaling method acknowledging that these fields are representing an average of the finer scale dynamics, and we believe representative for coastal waters. The outputs from climate models are typically provided at monthly resolution for the ocean (very rarely daily) and daily for the atmosphere and therefore we believe our approach best reflects how our methods most likely will be applied.

Three sets of particle simulations were forced by either GLORYS and ERA5 (dynamic reference), SD-GLORYS and SD-ERA5, or coarsened GLORYS and ERA5 to track the dispersal of water particles released along the coast of Aleutians East Borough over a period of 12 months (January 1^st^ to December 31^st^) for each of the years 2015 to 2020. For all simulation sets, ocean currents (*uo*, *vo)*, mixed layer depth (*mlotst*), and atmospheric wind (*u10*, *v10*) data were used as forcing for the particle tracking model. For each year between 2015 and 2020, a total of 20,000 particles were evenly spread (uniformly) and released within a radius of 30 km along a line along the coast of Aleutians East Borough (Alaska, USA) between -166.5^o^E, 54.00^o^N and -161.23^o^E, 56.06^o^N on January 1^st^ of each year. The choice of radius allows the random initial location of particles to be distributed across multiple grid cells (resolution ~ 5 × 5 km) within the GLORYS and SD-GLORYS current fields. Mean distance between particles were 528 m. The domain where particles were released was further divided into three regions Western, Central, and Eastern to allow for comparative analysis of areas with varying degrees of dominant currents and local dynamic variability (Supplementary Fig. 5). The time-step of the particle tracking was 1800 s for horizontal advection and 300 s for vertical mixing and convection. Monthly and daily datasets are interpolated both spatially and temporally using cubic interpolation, thereby enabling the estimation of oceanographic variables at any desired location and time. This interpolation capability, integral to the OpenDrift modeling framework, allows for seamless integration of multiple input datasets with diverse spatial and temporal resolutions, thus providing consistent and continuous forcing for Lagrangian particle tracking simulations^26^. The outputs of the model were stored for every 24 h.

### Evaluation and validation of downscaled vector currents

To our knowledge, downscaling vector variables that preserve both the scalar value and direction is not common and represents a novel, simplified approach for addressing the demand for high-resolution ocean currents and wind in e.g. mCDR applications on a global scale. The approach treats each vector component as a scalar and downscales these individually using the DQM methodology before combining them to produce a high-resolution vector product. Our assumption that this approach is viable and produces high-quality results requires downscaled currents and wind and particle distributions after tracking to be comparable to the outputs from the dynamic models.

Here, we validate our approach by first coarsening the high-resolution ocean vector fields from the GLORYS model to a reduced resolution of 1 × 1 degree longitude and latitude.

Coarsening was performed by averaging every 12 × 12 grid cell of the GLORYS dataset, reducing the dataset to a resolution typical of ocean climate model (CMIP6^6^) outputs. Next, we use the coarsened GLORYS dataset as input to our downscaling algorithm to produce a higher resolution dataset, to recreate the original high-resolution GLORYS dataset at a 1/12^th^ degree resolution. For the atmosphere, we coarsened the ERA5 surface wind vectors from 0.25° × 0.25° to 3.0° × 3.0° (typical resolution of CMIP6 atmospheric variables) before reverting the process and downscaling to higher resolution. The statistical downscaling methodology was trained on GLORYS and ERA5 data from 1993 to 2015, while the statistically downscaled outputs used to validate the approach were the years 2015–2020. This prevents using the same data to both train the model and skill test the outputs.

### Evaluation metrics

We applied four methods for evaluating how well the SD-GLORYS and SD-ERA5 models compared with the high-resolution GLORYS and ERA5 models. First, we used the Liu-mean^32^ skill score to quantify how well SD-GLORYS/SD-ERA5 recreates GLORYS/ERA5, next we calculated the Okubo-Weiss parameter to quantify strain and vorticity, and we calculated the Lagrangian Coherent structures (LCS) to analyze the underlying dominating flow pattern. Finally, we used the Bhattacharyya coefficient^33^ to evaluate the distributional overlap between particle trajectories forced with SD-GLORYS and SD-ERA5 and particle distributions forced with coarsened GLORYS and ERA5 relative to GLORYS and ERA5. These four approaches allowed us to understand the strengths and weaknesses of our downscaling approach of vector currents.

*Liu-mean skill score* the skill of the methodology in recreating the original dataset was quantified using the Liu-mean skill score^32^, which combines individual metrics, according to

$$LSE = 1 - \sqrt {\left( {\rho \alpha - 1} \right)^{2} + \left( {\beta - 1} \right)^{2} }$$

where α is the ratio of the downscaled model mean (SD-GLORYS/SD-ERA5) over the mean of the reference model (GLORYS/ERA5), β the ratio of the downscaled model and reference model standard deviation, and ρ was the spatial Pearson correlation coefficient between the downscaled model and reference. A skill score of 1 signifies a perfect alignment with reference data, while values less than 1 denote a reduced level of comparison between the downscaled model and reference. The first component of the skill score, which integrates the correlation coefficient and the ratio of standard deviations, assesses the deviation of the linear regression slope between the downscaled model and the reference values from 1. The second component serves as a non-dimensional indicator of the overall bias between the two datasets. In the extreme, a completely uncorrelated relationship between the two datasets would result in a value of 0 for the first component, leading to negative skill score values if there is any further deficiency in the second component. Similarly, if the standard deviations or the mean of the downscaled model were to reach twice that of the reference model, the skill score would become 0 or less, even in the presence of a perfect correlation between the downscaled and reference models.

The Okubo-Weiss parameter (W) is a fundamental diagnostic tool in oceanography that quantifies the relative dominance between strain and vorticity in fluid flows, allowing for characterization of the Eulerian flow topology:

$$W \, = \, s_{n}^{2} \, + \, s_{n}^{2} \, - \, \zeta^{2}$$

where $s_{n}^{2}$ is the normal component of total deformation (strain), s^2^ₛ is the shear component and ζ^2^ is relative vorticity. Here, we use the Okubo-Weiss parameter to quantify the spatial and temporal distribution and maintenance of vortical structures in the ocean, with the intention of comparing the consistency between the downscaled and original flow fields. High vorticity (*W* < 0) dominated regions can be associated with trapping of particles and thereby reducing their dispersion and represent a key feature of the flow field important to recreate in a downscaled flow field to realistically capture the dominating patterns. Regions with high strain (*W* > 0), deformation of the fluid flow enhances mixing and dispersion, causing particles to separate rapidly.

Lagrangian Coherent Structures (LCS) represent the underlying dominating flow pattern that partition ocean regions into dynamically distinct zones that undergo similar transport experiences^34–36^. LCS can be thought of as topographic maps of filamentation and transport boundaries in ocean circulation that can serve as barriers constraining fluid movement^36^. Using the ocean current vector flow field (*uo*, *vo*) we can calculate these LCS as the ridges of Finite-Time Lyapunov Exponent (FTLE) fields. In principle, the FTLE method estimates the maximum exponential separation rate of initially closely spaced fluid particles over a specific time interval in a chaotic flow field^35^. The FTLE can integrate particle movements both backwards and forward in time, with forward-time calculations identifying repelling LCS (divergence zones) and backward-time calculations revealing attracting LCS (convergence zones). As the LCS identifies general patterns of the flow field, ensuring that the SD-GLORYS vector field can reproduce the original GLORYS LCS is important for validating the assumption that dispersion of particles in downscaled fields is representative. The FTLE ($\sigma$) is defined as:

$$\sigma = \frac{1}{\left| T \right|}\ln f(x)\sqrt {\lambda_{max} }$$

where ${\lambda }_{max}$ is the largest eigenvalue of the Cauchy-Green strain tensor and T is the time. For this study we applied the FTLE method to monthly ocean velocity fields between 2015 and 2020 using GLORYS and SD-GLORYS data as input. We refer to existing literature for equations for in-depth details on calculating the FTLE^34–36^.

### Bhattacharyya coefficient

The Bhattacharyya coefficient^33^ (BC) quantifies the similarity between two distributions^37^. Here, BD was used to evaluate the relative improvement of using SD-GLORYS compared with the coarsened GLORYS relative to the baseline GLORYS simulation as forcing for particle tracking. BD was computed as:

$$\mathrm{BC}=(\sum_{i=1}^{N}\sqrt{({P}_{GLORYS}{P}_{model})})$$

Here, *P*_*GLORYS*_ represents the binned spatial distribution of trajectories simulated using GLORYS/ERA5 as forcing and *P*_*model*_ is the binned distribution of trajectories using either SD-GLORYS/SD-ERA5 or coarsened GLORYS/ERA5 as forcing. For this study, values close to 1 indicate a high while 0.0 suggests reduced degree of overlap. Values above 0.7 are generally considered to indicate good spatial agreement, while values below 0.6 suggest divergence in particle transport pathways.

## Results

### Validation downscaling

The downscaled CMCC-CM2-SR5 dataset showed that the bias correction for the training and the validation period created comparable mean current speeds *uo* and *vo* (Supplementary Figs. 1 and 2). The bias between GLORYS and original CMCC-CM2-SR5 and between GLORYS and bias-corrected CMCC-CM2-SR5 for both training period and validation periods showed consistent improvements after bias-correction for both eastward (*uo*, Supplementary Fig. 3) and northward (*vo*, Supplementary Fig. 4) velocities.

**Fig. 2:**
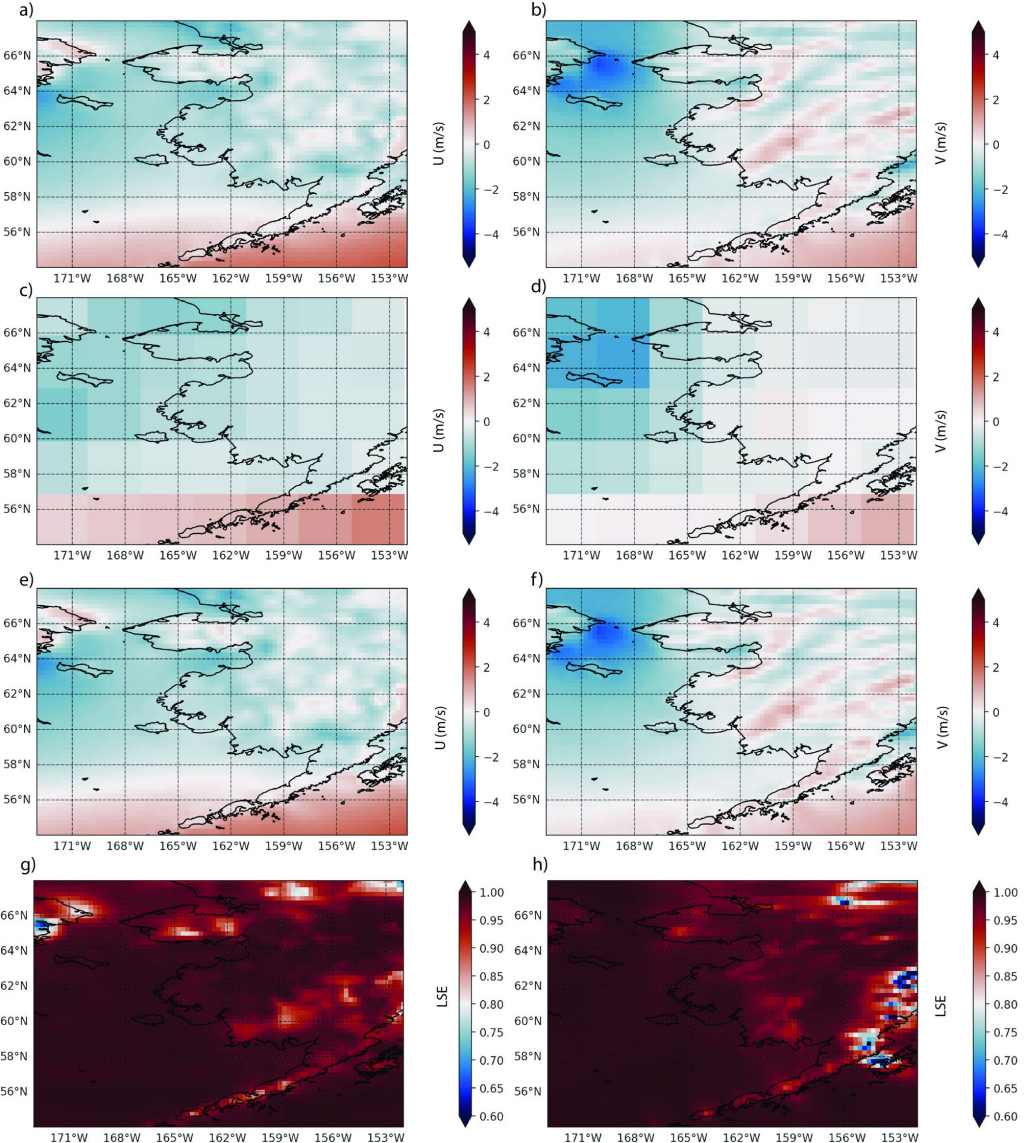
Top panel illustrates the daily original ERA5 wind speeds *u10* **a** and *v10* **b**. All rows show values averaged for the period 2015–01-01 to 2019–31-12. The second row presents the original ERA5 coarsened *u10* **c** and *v10* **d** wind speed to a resolution of 3° × 3°, which serves as input for the bias-correction and downscaling process. The third row displays the final downscaled (SD-ERA5) *u10* **e** and *v10* **f** wind fields created by downscaling the coarsened dataset. The final row shows the Liu-mean skill score between the original ERA5 and downscaled SD-ERA5 (3^rd^ row) for *u10* **g** and *v10* **h**. Figure created with Python v3.11.9 using modules Cartopy v0.23.0, Seaborn v0.13.2, and Matplotlib v3.9.2.

**Fig. 3:**
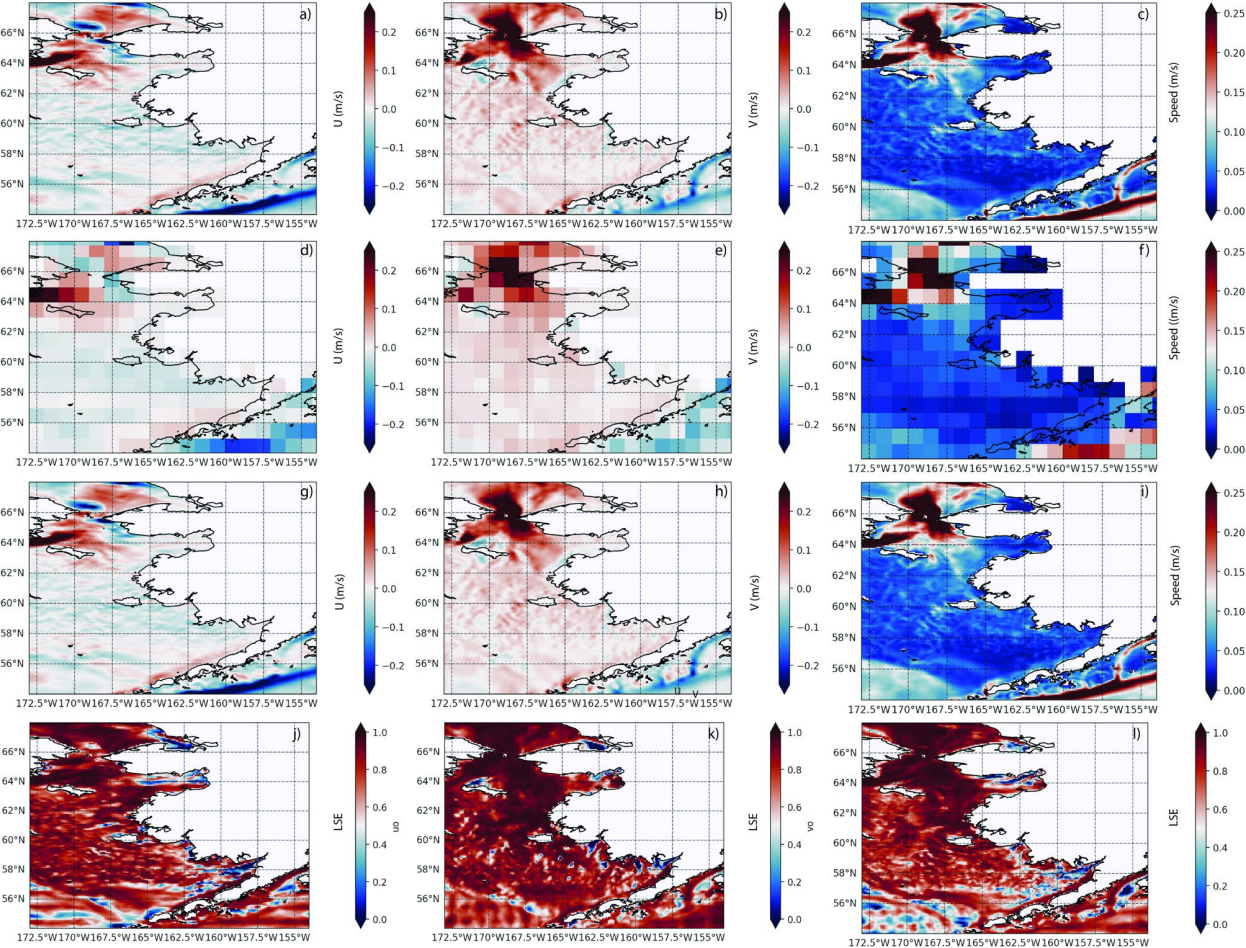
Top panel illustrates the monthly GLORYS ocean-current speed u (left column), v (middle column), and speed (right column) at 5 m depth. All rows depict values averaged for the period 2015–01-01 to 2019–31-12. The first row shows the GLORYS values **a**, **b**, **c**, while the second row **d**, **e**, **f** represents the same data coarsened to a resolution of 1° × 1°. The coarsened dataset serves as input for the downscaling process. The third row **g**, **h**, **j** displays the result of the downscaling (SD-GLORYS) that used the coarsened dataset as input. The final row shows the Liu-mean skill score between the original GLORYS and the equivalent downscaled high-resolution SD-GLORYS (3^rd^ row). Figure created with Python v3.11.9 using modules Cartopy v0.23.0, Seaborn v0.13.2, and Matplotlib v3.9.2.

The Liu-mean skill score of the downscaled ERA5 (SD-ERA5) surface wind components (Fig. 2) showed very high values with average 0.976 for *u10* and 0.975 for *v10*. The high skill dominated most of our focus region, indicative of a strong ability of the downscaling methodology to reproduce the original wind field. Liu-mean skill score for wind speed was on average 0.967. A few local areas had reduced skill (0.5–0.7), mainly located over land, while showing consistently very strong skill over the ocean (Fig. 2). The skill score for SD-GLORYS, focusing on both zonal (*uo*) and meridional (*vo*) current components at 5 m depth, suggested (Fig. 3) a more complex pattern compared to atmospheric wind. Overall, the average (2015–2020) skill for zonal speed was 0.81 and for meridional speed was 0.75 for 5 m flow fields. Combining both components, the average Liu-mean skill score for ocean speed was 0.75 (Fig. 3). These dynamic regions, such as the southwestern area of the Eastern Bering Sea, are often associated with strong transient eddy activities where downscaled features may be out of sync with observed features, causing low correlation in space and time. Regions dominated by consistently strong prevailing ocean current patterns and with lower variability showed consistently high skill (Fig. 3). For example, the Alaska Coastal Current region showed high skill scores (0.8–1.0), while moderate to high skill (0.4–0.7) dominated parts of the continental shelf regions. Lower skill scores (0.2–0.4) for both *uo* and *vo* appeared in some offshore areas, particularly in the southwestern portion of the domain. Reduced skill was associated with regions with high dynamic variability, relatively small velocity values, and more transient physical features.

### Okubo-Weiss (OW)

**Fig. 4:**
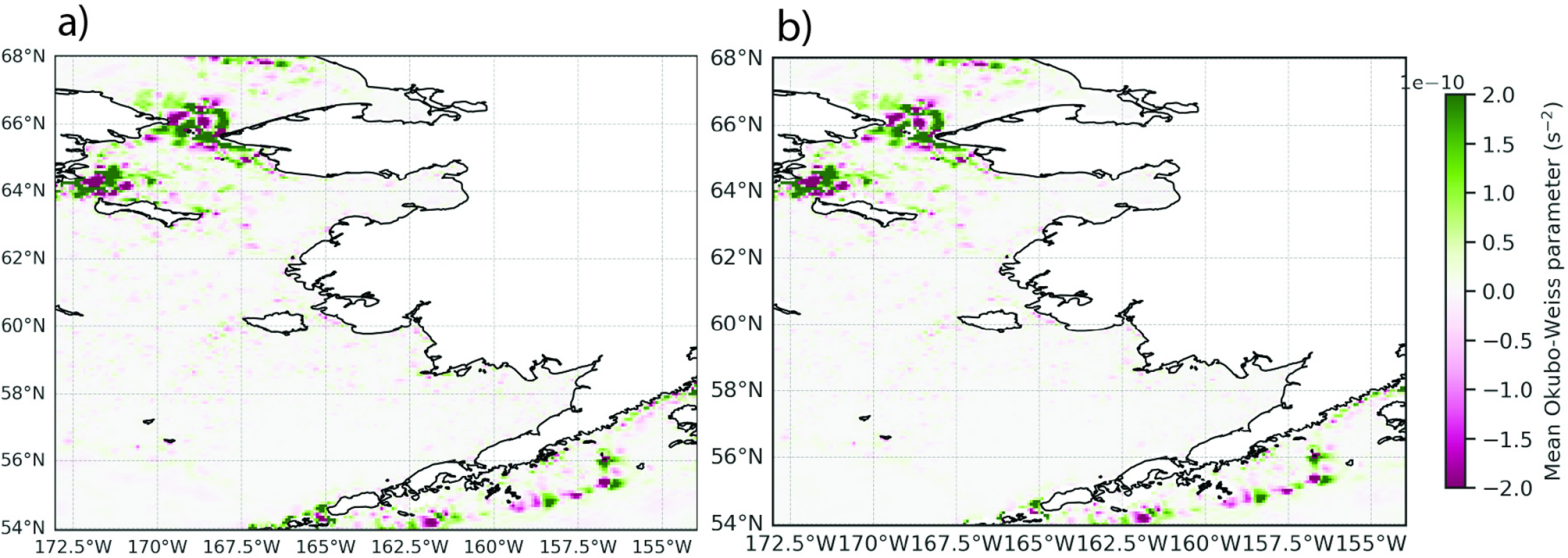
Comparison between the mean Okubo-Weiss parameter (s^−2^) for **a** GLORYS and **b** SD-GLORYS. Values were calculated for each month and year between 2015 and 2020 and averaged. Green positive values indicate permanent features while negative values (pink) indicate persistent eddies. Figure created with Python v3.11.9 using modules Cartopy v0.23.0, Seaborn v0.13.2, and Matplotlib v3.9.2.

Mean parameter values of OW showed high skill when comparing GLORYS and SD-GLORYS averaged for the period 2015–2020 (Fig. 4), suggesting that the dominant physical features are reproduced in SD-GLORYS. Permanent physical features as well as the resident eddies are realistically featured in the downscaled reproduction. In SD-GLORYS, the minimum OW values, indicative of eddies, for the surface waters showed significant variability over the 2015–2020 period, with values around 10^–9^ to 10⁻^8^ s⁻^2^ (Supplementary Fig. 5). The GLORYS currents showed occasional sharper minima which suggests periods of stronger vorticity-dominated flow or increased eddy activity at the surface during those times. The SD-GLORYS patterns are similar but with fewer and less pronounced minima. This suggests that the downscaled flow fields preserve temporal fluctuations but may smooth extreme variability. The monthly fraction of area with negative OW (indicative of rotational or eddy-dominated flow) varies between ~ 21.75% and 23.5% for GLORYS and 23.0%–24.3% for SD-GLORYS (Supplementary Fig. 6). This suggests that a larger area of the downscaled flow field is classified as surface eddies, potentially caused by statistical effects of downscaling. Overall, the results indicate that the flow field of the Bering Sea maintains a persistent pattern where less than one-fourth of the surface is dominated by rotational motion, with the remainder characterized by strain-dominated or transitional regimes.

### Lagrangian coherent structures

**Fig. 5:**
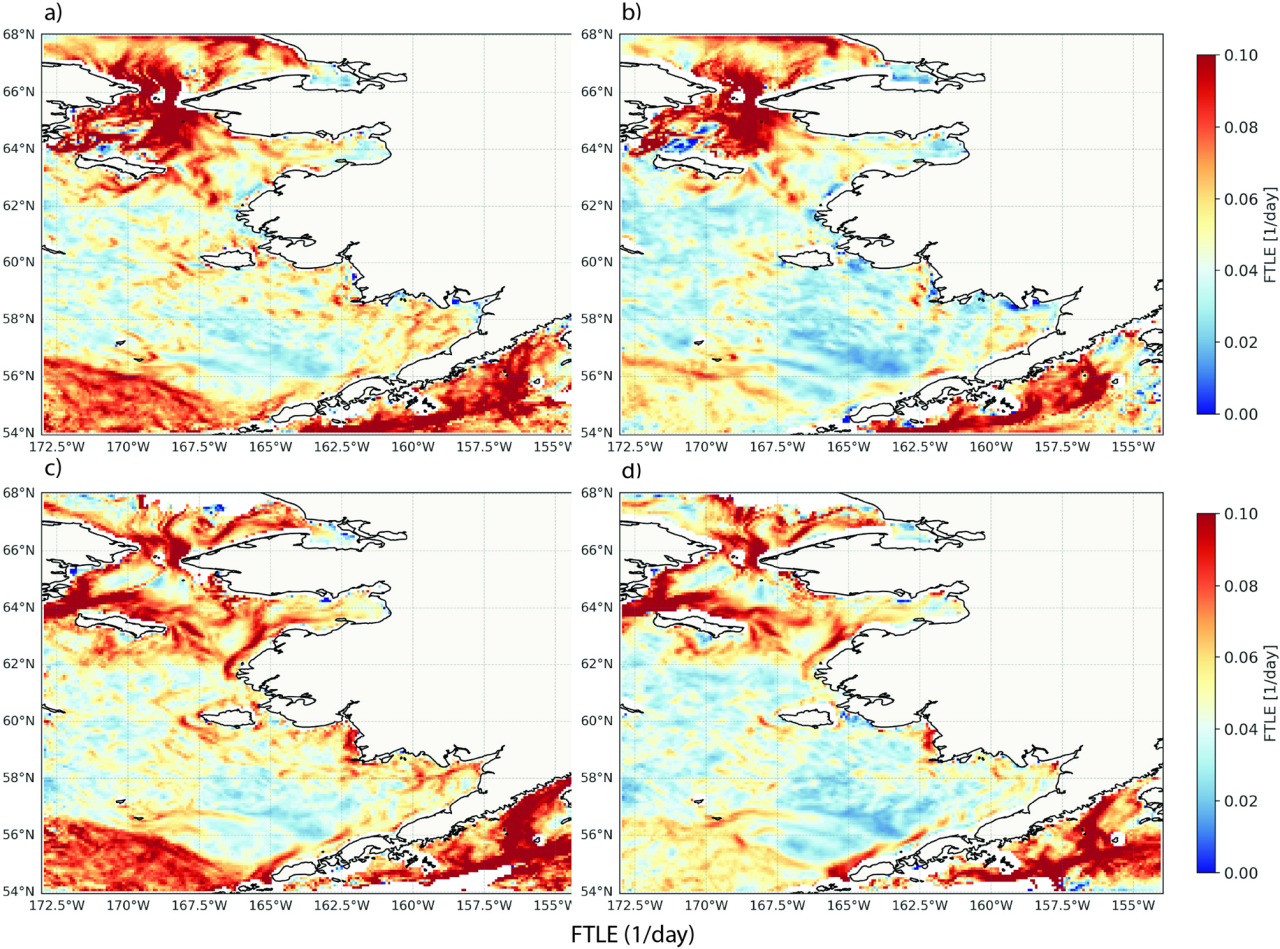
Comparison between the consistency of Lagrangian Coherent Structures identified using the Finite-time Laypunov Exponent (FTLE) methodology. The FTLEs (1/day) were calculated both backward **a**, **b** and forward **c**, **d** tracking the separation of water parcels for 15 days period for each month between 2015 and 2020 and then averaged. Left column **a**, **c** shows the original GLORYS model while the right column **b**, **d** shows SD-GLORYS (high-resolution downscaled coarse GLORYS). Figure created with Python v3.11.9 using modules Cartopy v0.23.0, Seaborn v0.13.2, and Matplotlib v3.9.2.

Figure 5 shows the FTLE fields revealed consistency in Lagrangian Coherent Structures (LCS) between GLORYS and SD-GLORYS covering the Bering Sea, the Gulf of Alaska and surrounding waters. The FTLE scale values (1/day) indicate the stretching of particle trajectories over 15-day integration periods and can be used to reveal coherent structures in the meso-scale ocean dynamics, important for analyzing transport behavior in time varying flow fields including shear, confluence and eddies.

*Backward-time* Backward FTLE reveals attracting areas where particles initially originating in different locations may accumulate due to convergent transport^38^. Both the GLORYS (Fig. 5a) and SD-GLORYS (Fig. 5b) models exhibited a prominent high-intensity FTLE ridge (red region) in the northwestern Bering strait (64–66°N, 168–165°W). This feature represents a significant convergence zone that persists in both models, suggesting it is a robust feature of the regional circulation. The preservation of this structure in the downscaled model indicates that the statistical downscaling process maintains this critical attracting mechanism. The shape and extent of this primary attracting structure showed remarkable consistency between models. In both cases, the structure extended southeastward from the northwestern corner with similar spatial dimensions. This consistency suggests that the downscaling process preserved not only the existence but also the geometry of major attracting features in the flow. While the general pattern was maintained, there were subtle differences in the intensity distribution within the attracting structure. The GLORYS model (Fig. 5a) appeared to have a slightly more intense and sharply defined boundary of the attracting region compared to the SD-GLORYS model (Fig. 5b), particularly in the deeper waters in the southwest corner of the Bering Sea. This could indicate some smoothing of gradient information during the downscaling process, though the principal features remain intact.

*Forward-time*: Forward FTLE analysis reveal areas where particles initially close together separate over time due to dispersion and divergence. The FTLE magnitude ranges (0.00–0.10 day^−1^) are similar between models, indicating consistent overall transport characteristics, with strong similarity in ridge continuity (Fig. 5c, d). The SD-GLORYS model captures the meso-scale features and the topographic constraints seen in the GLORYS model. The most notable difference is the southwestern part of the Eastern Bering Sea where the bathymetry is much deeper with steep gradients. There, the darker red regions visible in the GLORYS model (panel c) is a more fragmented and less pronounced pattern in the SD-GLORYS model (panel d). This suggests that the statistical downscaling process may not fully preserve the fine-scale repelling structures that organize flow divergence in the original model. Both models clearly depict the Alaska Coastal Current system through prominent positive FTLE ridges (red) along the coastlines in the forward-time calculations (Fig. 5c, d), although the SD-GLORYS is slightly weaker towards Kodiak Island. These ridges represent areas of strong stretching typical of boundary currents where the flow accelerates and diverges. The ridges appear particularly pronounced along the Alaska Peninsula (around 56°N-60°N, 156°W-153°W) and the northern Gulf of Alaska coastline.

### Validation of particle distributions

**Fig. 6:**
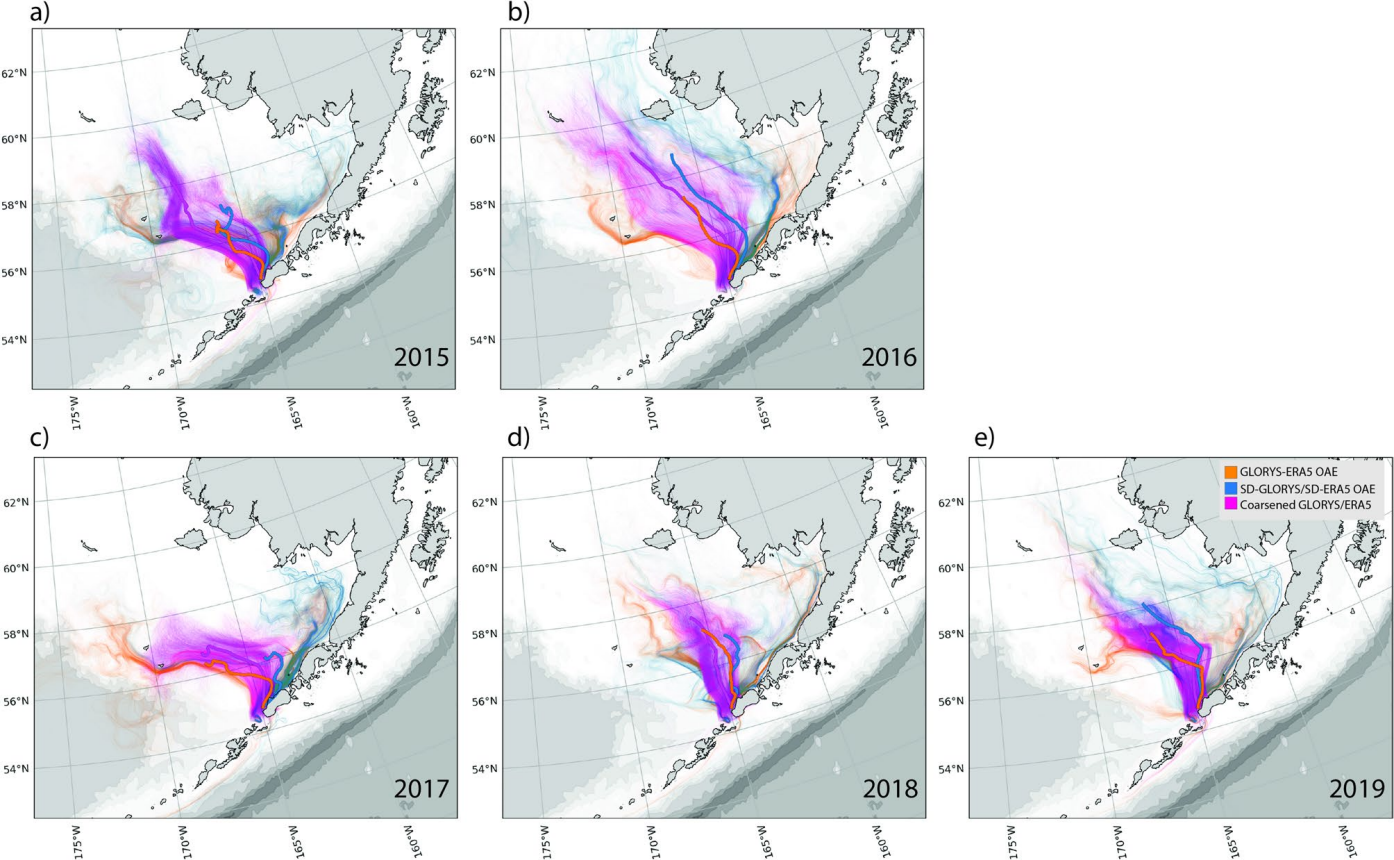
Comparison between individual and mean particle trajectories tracked over 12 months (January through December) for the years 2015–2019 using ocean current fields from the high-resolution SD-GLORYS/SD-ERA5 downscaled flow fields (blue), the original GLORYS/ERA5 (orange) reanalysis, and coarsened GLORYS/ERA5 (fuchsia) for Western release sites along the coast of the Aleutians East Borough, Alaska. Figure created with Python v3.11.9 using modules Cartopy v0.23.0, Seaborn v0.13.2, and Matplotlib v3.9.2.

**Fig. 7:**
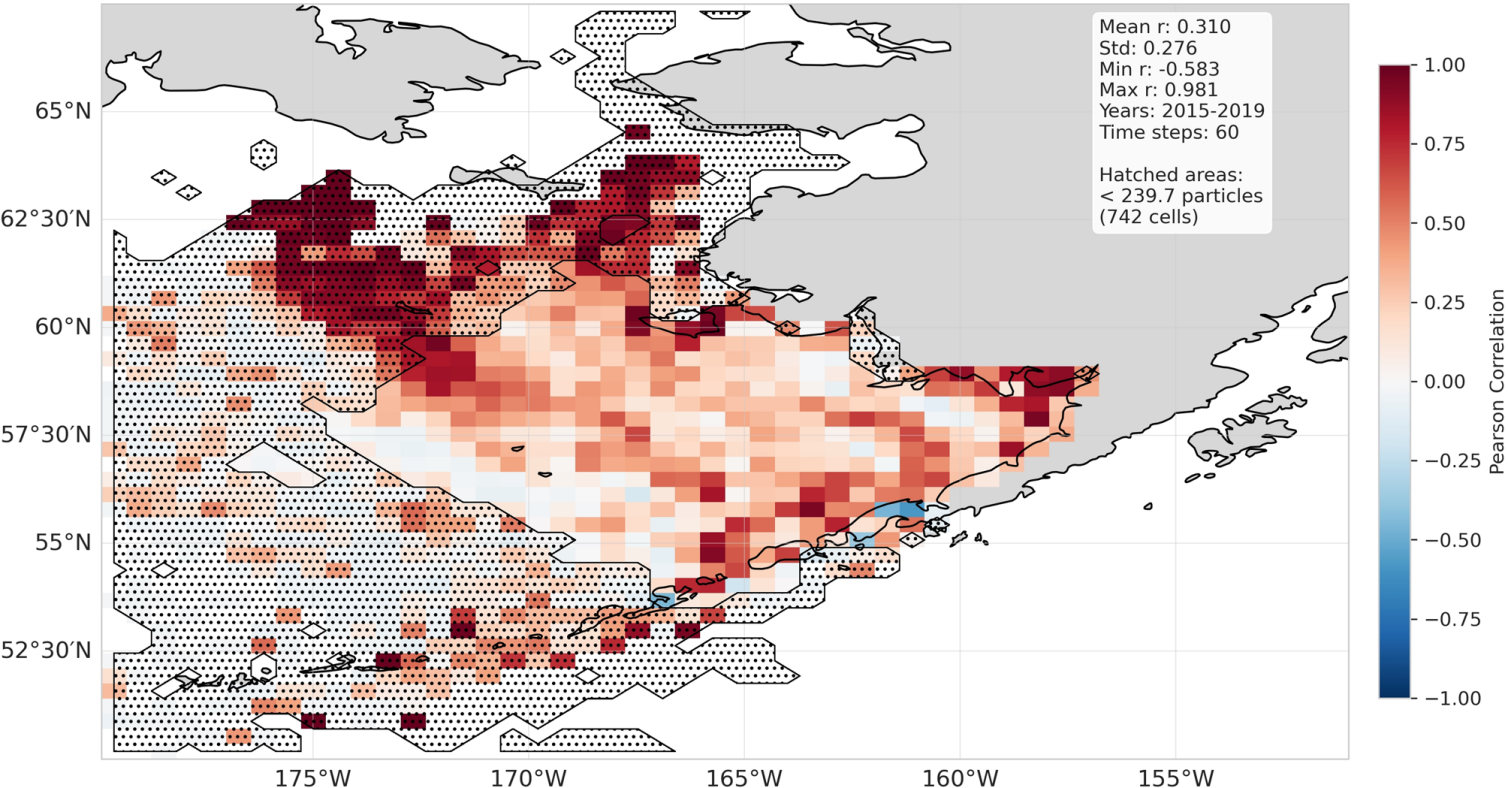
Temporal correlation of particle distributions between GLORYS/ERA5 and SD-GLORYS/SD-ERA5 simulations over the years 2015–2020. Correlations were calculated by binning the distributions for each month for each year 2015–2020 using a 50 × 50 grid. Concentrations within each cell were then correlated using Pearson correlation to identify how the model outputs differ in their temporal dynamics. Hatched areas are cells containing less than 0.01% of the total particles (2,396,796) summed over 5 years. Figure created with Python v3.11.9 using modules Cartopy v0.23.0, Seaborn v0.13.2, and Matplotlib v3.9.2.

**Fig. 8:**
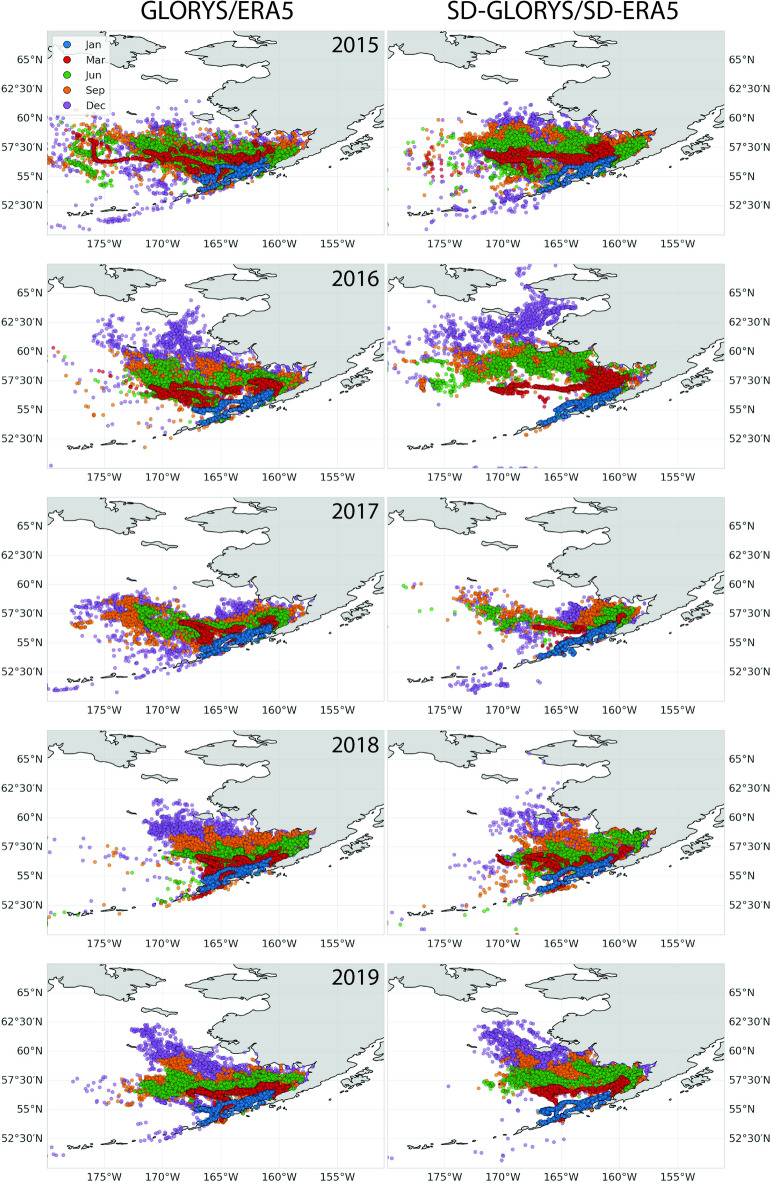
Spatial particle distributions at five different times during simulations. On the 15^th^ of each of the months January (blue), March (red), June (green), September (orange), and December (magenta) the distribution of particles is shown with a distinct color. The left column shows the GLORYS/ERA5 results while the right column shows the SD-GLORYS/SD-ERA5 simulation results for each year 2015–2020. All particles were released along the coast of the Aleutians East Borough, Alaska, USA on January 1^st^ of each year and tracked for exactly 1 year. Figure created with Python v3.11.9 using modules Cartopy v0.23.0, Seaborn v0.13.2, and Matplotlib v3.9.2.

The three particle distribution sets, forced by either GLORYS/ERA5, SD-GLORYS/SD-ERA5, or coarsened GLORYS/ERA5 for each individual year 2015–2020, were compared and presented as individual and average particle tracks (Fig. 6, Supplementary Figs. 8 and 9). These results were also presented as spatial–temporal correlations for the 2015–2020 distributions (Figs. 7–8). Geographically, skill varies significantly across the domain, with some regions consistently showing higher performance than others. The temporal correlation of 5-years of particle simulations compares SD-GLORYS/SD-ERA5 with GLORYS/ERA5 across time at fixed locations (Fig. 7), and we find a mean correlation after 1 year of tracking to be r = 0.31. Even if both models agree on general spatial patterns, they may disagree on the specific timing and magnitude of particle arrivals at each location. Overall, the 5-year spatial correlation between the particle distributions on the 15th of January (15 days since release), March, June, September, and December (350 days since release) revealed a spatially averaged Pearson correlation of r = 0.86, 0.75, 0.59, 0.59, and 0.57 (Fig. 8). The mean across the 5 years for the full year of simulation was r = 0.62. The separation distance across all particle distances between all individual GLORYS/ERA5 particles compared with all SD-GLORYS/SD-ERA5 showed that the overall spatial distributions, the distances between each individual particle trajectory across all the others, after 1 year of drift for the two realizations were comparatively similar (not shown). Over the period 2015–2020, the average distance travelled by a particle was 269.9 km and the maximum distance travelled was 2887.4 km. The comparisons demonstrate that statistically downscaled models provide physics that closely resembles the original, which when used in particle tracking realistically disperses the particles. Given that each particle trajectory path is exposed to sub-grid stochastic diffusion the resemblance between trajectories forced by dynamic or downscaled models is not expected to be identical, except on average across all trajectories.

The Bhattacharyya coefficient score was utilized to quantify the improvement achieved by forcing particle tracking simulations with SD-GLORYS in comparison to coarsened GLORYS relative to the GLORYS fields. This assessment examined whether large-scale forcing alone suffices for realistic particle trajectory prediction, or if statistical downscaling is necessary to enhance model performance. Results showed an overall substantial increase in skill when SD-GLORYS was used as the forcing dataset, compared with coarsened GLORYS (Table 1). While the model domain is primarily influenced by the dominant current systems present in both model configurations, the absence of detailed coastal features in the coarsened data is apparent (Fig. 6, Supplementary Figs. 7 and 8). For example, particle tracks along the Aleutian Islands reveal that coarse-resolution trajectories remain predominantly offshore, whereas only the original GLORYS and SD-GLORYS simulations permit particle passage southwestward through the Unimak pass (see Fig. 6).

**Table 1:** The Bhattacharyya Coefficient measures spatial distribution similarity between model outputs and the reference GLORYS simulation. Values range from 0 to 1, with higher values indicating better agreement with the reference simulation.

Year	Region 1 West SD-GLORYS/SD-ERA5	Region 1 West COARSENED GLORYS/ERA5	Region 2 Central SD-GLORYS/SD-ERA5	Region 2 Central COARSENED GLORYS/ERA5	Region 3 East SD-GLORYS/SD-ERA5	Region 3 East COARSENED GLORYS/ERA5
2015	0.87	0.69	0.75	0.62	0.79	0.67
2016	0.75	0.80	0.61	0.59	0.55	0.49
2017	0.81	0.71	0.85	0.75	0.70	0.64
2018	0.88	0.70	0.79	0.81	0.44	0.60
2019	0.82	0.53	0.69	0.87	0.50	0.51
Regional Average	0.83	0.69	0.74	0.73	0.59	0.58

Performance analysis reveals that SD-GLORYS/SD-ERA5 high-resolution approach is particularly advantageous in western regions, such as the Western region, where it outperforms coarsened GLORYS by an average of 0.14 points (0.83 vs 0.69), a substantial 20.4% improvement, underscoring the importance of finer-scale resolution for accurately capturing particle dispersion in areas with complex coastal dynamics (Table 1). SD-GLORYS/SD-ERA5 also demonstrates greater temporal consistency for the Western region, suggesting that high resolution yields a more robust representation of inter-annual variability. The particle trajectory visualizations for the western region (Fig. 6) clearly illustrate how both approaches capture the general southwestward transport pattern along the Alaska Peninsula, but with varying degrees of spatial spread and pathway accuracy. The tighter agreement of SD-GLORYS/SD-ERA5 particles with the reference GLORYS/ERA5 trajectories (shown in blue) reflects the method’s ability to preserve mesoscale circulation features that are smoothed out in coarsened products.

The Central region exhibited the most variable performance across years for both approaches. The coarsened forcing showed particularly high temporal variability (standard deviation = 0.120 across years), with Bhattacharyya coefficients ranging from 0.59 to 0.87. The SD-GLORYS/SD-ERA5 results also showed considerable variability (standard deviation = 0.092), ranging from 0.61 to 0.85. The near-equivalent performance in this region suggests that simple coarsening may adequately capture particle transport in areas where large-scale circulation patterns dominate over fine-scale bathymetric steering or mesoscale eddies. The central release sites appear to be in a transitional zone where neither downscaling nor coarsening provides a clear systematic advantage.

In regions with highly complex dispersion regimes, such as Eastern region, SD-GLORYS/SD-ERA5 maintains a slight edge (0.59 vs 0.58), although both models show limited effectiveness. Both methods frequently produced Bhattacharyya coefficients below the 0.6 threshold, indicating divergence from the reference simulation. The particle trajectory patterns in the eastern region reveal complex, bifurcating pathways with some particles transported southwestward along the peninsula while others are advected. This complex circulation regime, likely influenced by the interaction of the Alaska Coastal Current with bathymetric features near Unimak Pass, presents challenges for both downscaling and coarsening approaches. This is also an area where the downscaling underestimated the LCS, suggesting dynamic features were not fully resolved in SD-GLORYS/SD-ERA5.

Across all 15 simulation scenarios (three regions × five years), the SD-GLORYS/SD-ERA5 forcing achieved a mean Bhattacharyya coefficient of 0.720 ± 0.133, compared to 0.665 ± 0.109 for the coarsened GLORYS, representing a statistically meaningful improvement in spatial overlap with the reference GLORYS simulation. This indicated that higher resolution generally enhances performance, but that in areas with particularly dominating flows the benefits are reduced as even coarse resolution models capture such flows. In areas with strong coastal currents, eddies, and localized dynamics, the high-resolution downscaled model is critical for realistic simulations.

## Discussion

Dynamic ocean models are critical for our understanding of non-linear oceanic physical processes shaped by the local bathymetry and can help us understand how the physical environment drives ecological interactions and connectivity, carbonate chemistry, biological production and dispersal through ocean transport and mixing. For example, models can inform us on future efficiency and dispersal of individual water parcels of enhanced alkaline water parcels as in mCDR interventions^39^, estimating dispersal of fish larvae from important spawning grounds^1^, or tracking the interaction of oil spills with fish eggs^40^. However, dynamic models face operational limitations in geographic scope and replicability. While these models effectively represent oceanic features, computational demands restrict their ability to generate multiple ensemble simulations needed for uncertainty quantification and climate risk assessment. This limitation is especially severe when downscaling global climate models to regional scales, where processing capacities constrain simulation repetition. To solve this problem, new approaches for downscaling are emerging such as using latent diffusion models for enhancing the resolution of atmospheric reanalysis^14^ or convolutional neural network predictions of dust aerosols over Sahara^41^. A greater number of applications suggests both a growing reliance on downscaling as a tool and a greater demand for it. Still, statistical downscaling inherently depends on the availability of historical dynamic models or high-resolution observations as a foundational baseline^15,16^. As demonstrated here, historic statistical relationships between coarse (1^o^ × 1°) and finer (0.0833^o^ × 0.0833°) scale hydrodynamic fields can be used to recreate finer scale fields over novel temporal domains with sufficient fidelity to simulate transport patterns that are broadly similar to those from direct fine scale field simulations. This efficiently allows high-resolution dynamic models that cover a restricted historic period to be reused and extended to broader use by downscaling climate models or coarser resolution hindcast models, if the period covered by the statistical model represents the dynamic variability of the area.

This study showed that the downscaled ocean current and atmospheric wind were skillful in replicating the physical features from the original high-resolution dynamic model, but there are also regions with reduced skill and some differences in fine-scale features, such as the Eastern region of the Bering Sea. A recent study^14^ used a generative deep learning model to produce high-resolution surface temperature and wind fields for terrestrial Italy, and the authors argue that downscaling vectors is more challenging due to the combination of direction and scalar value, equivalent to what we find in this study. Even so, replicating the atmospheric fields in this study showed very high skill, higher than for the ocean, which is likely an effect of the difference in scale between the Rossby radius of deformation for atmosphere versus ocean. The disparities in scale in Rossby radius fundamentally determines the characteristic size of eddies and circulation features within each realm. The first baroclinic Rossby radius in the ocean decreases from over 200 km near the equator to less than 10 km at latitudes beyond 60°, creating inherent resolution requirements for capturing mesoscale processes^42^. This latitudinal compression contrasts sharply with the atmosphere’s Rossby radius of 1,000–2,000 km in mid-latitudes, explaining why statistical downscaling achieves higher skill in atmospheric applications with coarser baseline inputs. Because of this, using statistical downscaling to resolve detailed physical features like mesoscale eddies at high-latitudes generally requires a higher-resolution baseline for the ocean than for the atmosphere.

The Okubo-Weiss analysis revealed a complex but structured flow field with clear implications for particle transport, which were comparable between the GLORYS and SD-GLORYS flow fields (Fig. 4). These results suggest that the downscaling method was able to resolve many dynamical features like mesoscale eddies at high latitudes. The temporal stability in rotational area percentage, combined with variability in minimum values, suggests a dynamic equilibrium between coherent structures and background flow, where negative OW values are associated with vorticity or eddies. For Lagrangian applications, these patterns would create a mosaic of retention zones (negative OW) and mixing/transport corridors (positive OW) that would fundamentally shape transport pathways in the region of interest. For regional models, and their downscaled equivalents, it is important to capture mesoscale eddies as these are of key importance for the movement of energy from large to smaller scales.

Identifying LCS using the FTLE approach provides deep insights into complex oceanic phenomena like shear, convergence, eddies, and oceanic fronts, complementing the Eulerian Okubo-Weiss analysis. The use of LCS has been applied to study coastal ocean mixing^43^, understanding mesoscale and sub-mesoscale circulation^44^, and applications for marine species distribution is increasing^45^. Here, we applied the FTLE approach to analyze if the structures of the original GLORYS flow field were regenerated when the field was downscaled from its coarser version (SD-GLORYS). The comparison revealed that while SD-GLORYS captured the major LCS features present in GLORYS, there are notable differences in their representation. This suggests that the downscaling process may not fully preserve all dynamical features, particularly those related to meso-scale processes, which is particularly associated with small-scale processes associated in areas of high vorticity and steep bathymetric features such as the Aleutian basin at the southwestern corner of the Eastern Bering Sea. This was also found in the Bhattacharyya skill evaluation. The differences observed between models highlight challenges in maintaining these intricate structures during downscaling which in the future could potentially be improved using more complex artificial intelligence.

The strong consistency in backward-time FTLE fields between models indicates that the downscaling process preserved the major attracting mechanisms in the regional circulation. This further suggests that processes related to convergence, such as downwelling, frontal accumulation, and material aggregation, are represented in the downscaled model. The differences observed in the forward-time FTLE fields indicate that the downscaling process modified the representation of transport barriers in the flow. This could have implications for applications focused on material transport, such as pollutant dispersion, biological connectivity, or heat transport, as these processes are influenced by the presence and strength of repelling structures. The analysis suggests a scale-dependent preservation of LCS in the downscaling process. Large-scale features, such as dominating persistent current and frontal systems, particularly in the attracting structures, appear well-maintained, while smaller-scale features and the intensity of transport barriers show greater discrepancies.

Our skill assessment of particle distribution from the initial starting point using either GLORYS-ERA5 or SD-GLORYS/SD-ERA5 revealed that overall, the distributional patterns are comparable across years, but that small differences in the flow fields can escalate (Fig. 6, Supplementary Figs. 7 and 8). Still, this is not surprising given the chaotic turbulent flow fields where small differences in spatial distance can quickly grow. In addition, the threshold of 1 year for particle tracking represents a long integration period during which small initial velocity errors can compound. Studies in the Gulf of Mexico have identified critical separation times (ranging from 16 to 30 days) after which particle dispersion follows asymptotic power law growth independent of initial separation distance^46^. Before reaching this critical time, dispersion showed strong spatiotemporal dependence with distinct similarity regimes. This transition point indicates when the particles’ separation becomes dominated by larger-scale features rather than initial conditions. The spatiotemporal correlations of the dispersal of particles between the two sets of trajectories at specific times through the years remained consistently high (r = 0.61–0.75) for the first year of drift. This could be a result of our ability to downscale the prevalent, strong dynamic oceanographic features in the Bering Sea where our release point was located, while other source locations may have been more challenging. For example, we did see that areas where vorticity dominates, the variance is high, and the velocity values are relatively low, it can be challenging to downscale the flow field, suggesting there is room for improvement to our methodology. Analysis using the Bhattacharyya skill score demonstrated that particle trajectories forced with the coarsened GLORYS fields exhibited reduced skill compared to those forced with the statistically downscaled GLORYS (SD-GLORYS). This outcome is expected, as the coarsened model does not resolve mesoscale features but instead captures only the dominant and persistent features of the ocean currents. Previous studies have demonstrated that the absence of mesoscale eddies in coarse-resolution ocean models significantly degrades Lagrangian particle tracking performance, as these models fail to capture both the turbulent dispersion and the correct mean flow fields that eddies generate through rectification effects^47,48^. The importance of resolving mesoscale features for accurate particle trajectory modeling has been well-established across diverse applications, from tracking marine organism dispersal to simulating sediment transport^49^. These findings illustrate that employing statistically downscaled forcing fields for particle tracking significantly enhances the capacity of the model to represent realistic trajectory behavior.

### Balancing computational efficiency with accuracy in marine modeling

This study presents an alternative to traditional dynamic ocean models like ROMS and NEMO, addressing a critical bottleneck in marine intervention modeling by using downscaled vector currents. Statistical downscaling has been applied to vector fields before, particularly in enhancing surface wind predictions in relation to wind farming^50^, but to our knowledge it has not been applied to ocean currents. A statistical approach that combines downscaled ocean currents with Lagrangian particle tracking, provides a computationally efficient framework that maintains high fidelity in predicting dispersion patterns. Several Ocean Alkalinity Enhancement (OAE) studies have used Eulerian approaches to understand the efficiency of an intervention^51–53^, however these studies are either limited to a regional geography, or the level of detail is using coarser resolution physics (e.g., 1^o^ × 1°) and does not realistically represent coastal dynamics. A recent study^54^ used a dynamic ROMS model to track a hypothetical OAE intervention where alkalinity was added continuously into the waters of the Unimak pass, while the impacts on the carbonate system was monitored for 10 years. However, the regional constraints of the model domain prevented an understanding of the wider geographic consequences. A statistical downscaling approach solves this problem as the solution is scalable to global level if necessary. Further, this capability parallels the advantages of statistical downscaling in climate science, where improved spatial and temporal resolution makes data more useful for local and regional analyses. For monitoring marine interventions like OAE, where a significant portion of uptake of CO₂ can occur far from the initial intervention and the actual signal of the intervention rapidly falls below the detection limit of observational networks^39^, this modelling framework with geographic flexibility is particularly valuable, as is the ability to run multiple climate scenarios to better understand potential ecological impacts under different future conditions. In this study we did find that the skill was particularly high in regions dominated by prevailing ocean currents, while areas with high eddy activity show reduced predictive capacity. This trade-off between computational efficiency and localized accuracy represents an important consideration for various tracking applications beyond carbon sequestration efforts.

The Liu-mean skill score assessment revealed that detrended quantile mapping provided an effective method for downscaling ocean current fields in Alaskan waters, with particularly strong performance in coastal and shelf regions. The evaluation demonstrated that this statistical downscaling approach can reproduce high-resolution current patterns from coarser inputs in many regions, though with some spatial variation in performance. This has important implications for ocean modeling applications in regions where high-resolution data may be unavailable or computationally prohibitive to generate directly. A similar finding in a recent study where the authors used stochastic-deterministic downscaling to resolve sub-meso scale physics, concluded that the downscaled model performance showed high skill when compared to a dynamic model, and was much more computationally efficient^55^. The DQM mapping technique applied in this study, demonstrated strong capability in reproducing an original high-resolution field from coarsened data, with skill scores averaging above 0.75 across the domain. We also found that the technique performed exceptionally well in regions of oceanographic importance, including coastal currents and shelf regions where accurate representation of currents is critical for applications like fisheries management and coastal planning. The high Liu-mean skill scores for *uo* and *vo* components and speed (0.75), suggests that the vector directional integrity during the downscaling process was maintained. The results captured many key physical features of both large and meso-scale phenomena such as eddies, however some areas with reduced skill indicate that the methodology could be enhanced in future work to improve the outcome, particularly in regions of high variability. Our choice of using DQM as the downscaling methodology was partly based on its ability to preserve mean climate signal from original simulation, the projected trend, and overall projected changes, but could be criticized for not necessarily preserving changes in the tails of the distribution (extremes)^21,25^. This limitation becomes increasingly more important for longer projection periods, with near-term projections (e.g., 15 years) experiencing less impact compared to century-scale projections extending to 2100^24^. The main reason for the potential errors in the extremes is due to the stationary, or time-invariant, assumption of the DQM method where we assume that the bias identified during training remains for the future^56,57^. This assumption can break down during abrupt climate transitions where the climate system crosses critical thresholds, or physical relationships underlying the bias correction such as changes in land-surface feedbacks^58^. Several new downscaling techniques have recently been developed such as combining statistical downscaling with hybrid machine learning. One approach significantly improved the performance of rainfall predictions by using a combined approach of artificial neural networks with principal component analysis^59^. Another study^14^ used deep learning as part of the statistical downscaling combined with observations to improve the fine-resolution downscaling of atmospheric temperature, suggesting that combining different techniques can overall improve the simulated sub-mesoscale physical features.

## Conclusion

This study advances the methodology for longer-term particle tracking in marine environments by introducing a statistical downscaling approach that combines the rigor of dynamic models with high computational efficiency and geographic flexibility. Overall, we find that the statistical downscaling of atmospheric wind and ocean currents, both speed and direction, showed high skill suggesting that the physical features of the dynamic ocean variability is well represented. In a few areas with high variability and low current speeds we found reduced skill, suggesting that the methodology can be improved. It is important to keep in mind that this case study for the Bering Sea may not be representative for regions where it may be particularly challenging to downscale physical ocean dynamics and further research may be warranted for global applicability. In general, the particle tracking using GLORYS/ERA5 compared with SD-GLORYS/SD-ERA5 showed strong comparison indicative of downscaled products able to realistically represent the overall transport of water parcels, which is essential for being used in marine applications such as mCDR. The combination of statistically downscaled current fields with Lagrangian particle tracking methodology employed in this study has significant potential for adaptation to a variety of marine tracking challenges, particularly if a requirement is looking into the future using downscaled ocean and atmosphere current fields.

## Supplementary Information

Below is the link to the electronic supplementary material.

Supplementary Material 1
