# Supplementary material for: Statistical downscaling reproduces high-resolution ocean transport for particle tracking in the Bering Sea
Source: Sci Rep. 2026 Feb 4;16:7290. doi: 10.1038/s41598-026-37904-1 (PMC12923551; doi:10.1038/s41598-026-37904-1)
Supplement: Supplementary file 1 — Supplementary Material 1 [file 41598_2026_37904_MOESM1_ESM.pdf]

Supplementary info

Statistical downscaling reproduces high-resolution ocean transport for particle tracking in the Bering Sea

Trond Kristiansen[1,2], Jordan Miller[2], and Momme Butenschön[3]

[*1]Farallon Institute, Petaluma, California, USA
[2]Actea Inc, San Francisco, California, USA
[3]CMCC Foundation - Euro-Mediterranean Center on Climate Change, Italy

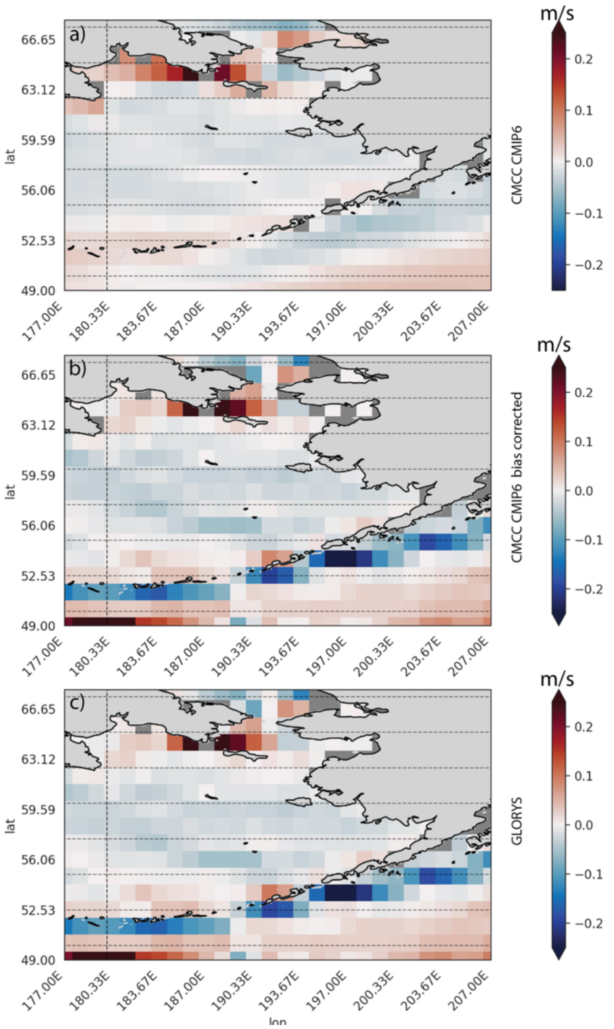

Supplementary figure 1: Mean (2015-2020) eastward ocean velocity (*uo* [m/s]) at 5 m depth shown for a) coarse resolution CMCC-CM2-SR5 CMIP6 model outputs, b) bias corrected downscaled outputs, c) GLORYS ocean reanalysis. Figure created with Python v3.11.9 using modules Cartopy v0.23.0, Seaborn v0.13.2, and Matplotlib v3.9.2.

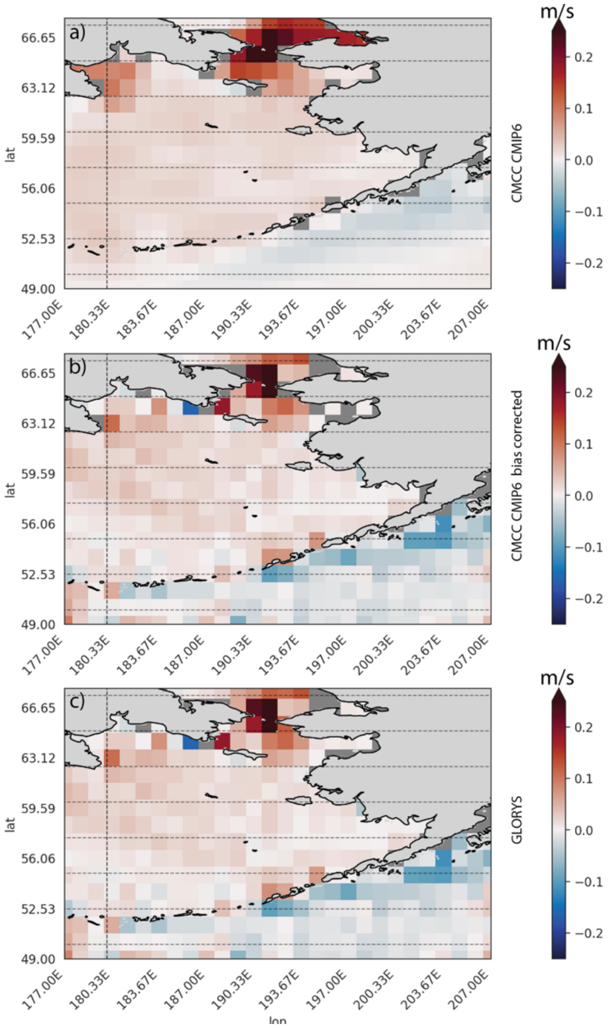

Supplementary figure 2: Mean (2015-2020) northward ocean velocity (*vo* [m/s]) at 5 m depth shown for a) coarse resolution CMCC-CM2-SR5 CMIP6 model outputs, b) bias corrected downscaled outputs, c) GLORYS ocean reanalysis. Figure created with Python v3.11.9 using modules Cartopy v0.23.0, Seaborn v0.13.2, and Matplotlib v3.9.2.

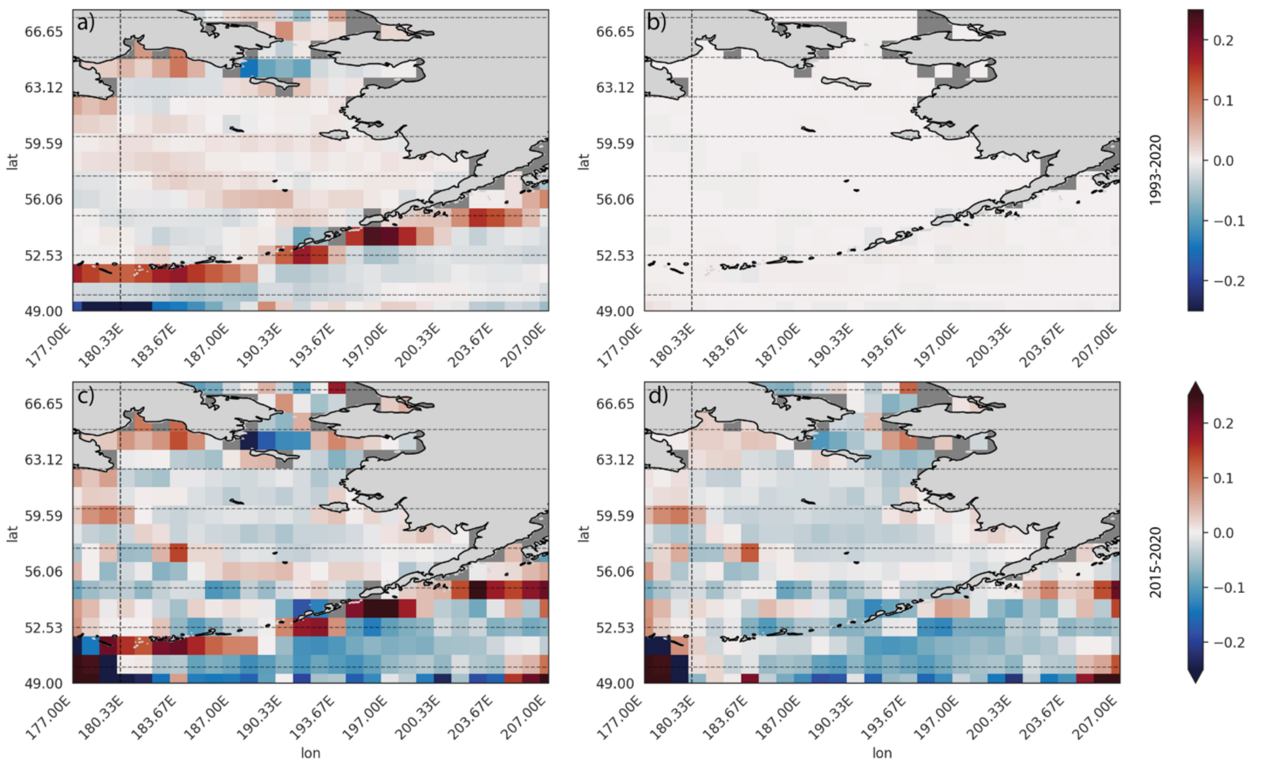

Supplementary figure 3: Bias of eastward velocity (*uo*) between a) CMCC-CM2-SR5 and GLORYS ocean reanalysis and b) bias corrected CMCC-CM2-SR5 for the training period 1993-2015 and bias between c) CMCC-CM2-SR5 and GLORYS ocean reanalysis and d) bias corrected CMCC-CM2-SR5 for the validation period 2015-2020. Figure created with Python v3.11.9 using modules Cartopy v0.23.0, Seaborn v0.13.2, and Matplotlib v3.9.2.

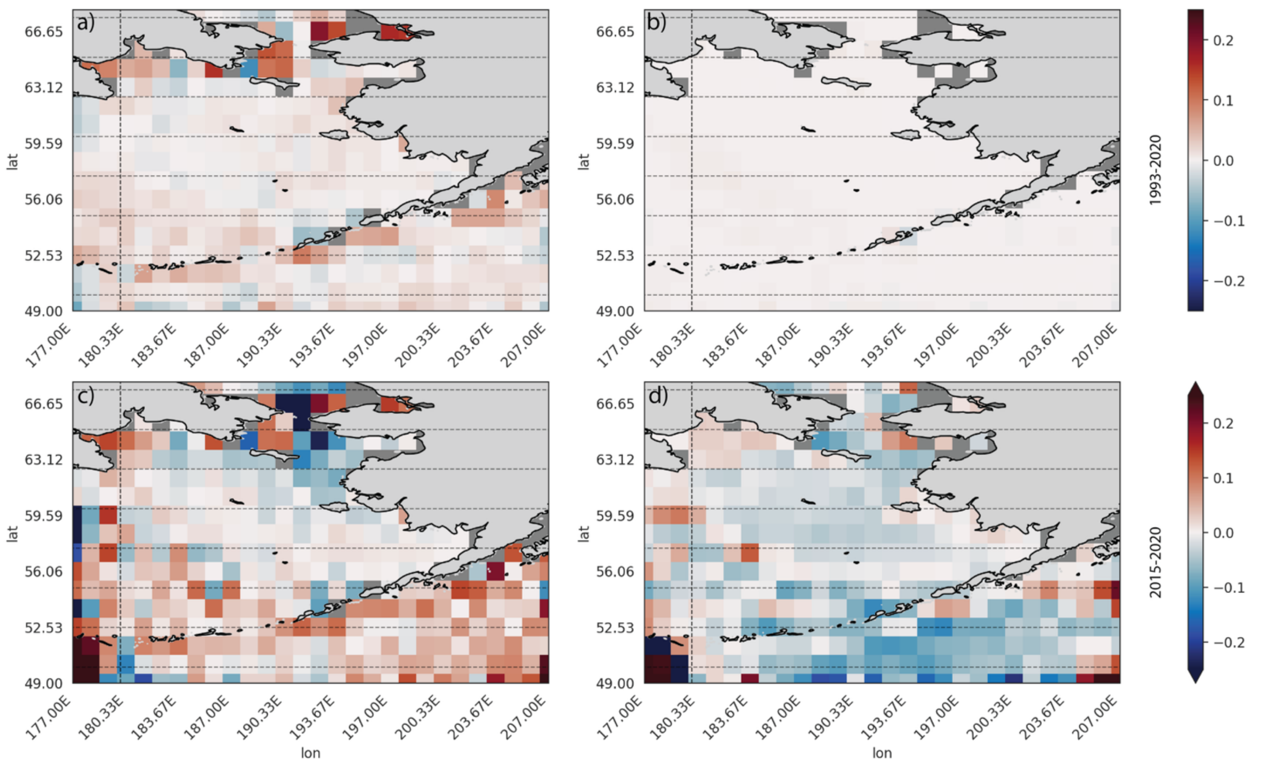

Supplementary figure 4: Bias of northward velocity (*vo*) between a) CMCC-CM2-SR5 and GLORYS ocean reanalysis and b) bias corrected CMCC-CM2-SR5 for the training period 1993-2015 and bias between c) CMCC-CM2-SR5 and GLORYS ocean reanalysis and d) bias corrected CMCC-CM2-SR5 for the validation period 2015-2020. Figure created with Python v3.11.9 using modules Cartopy v0.23.0, Seaborn v0.13.2, and Matplotlib v3.9.2.

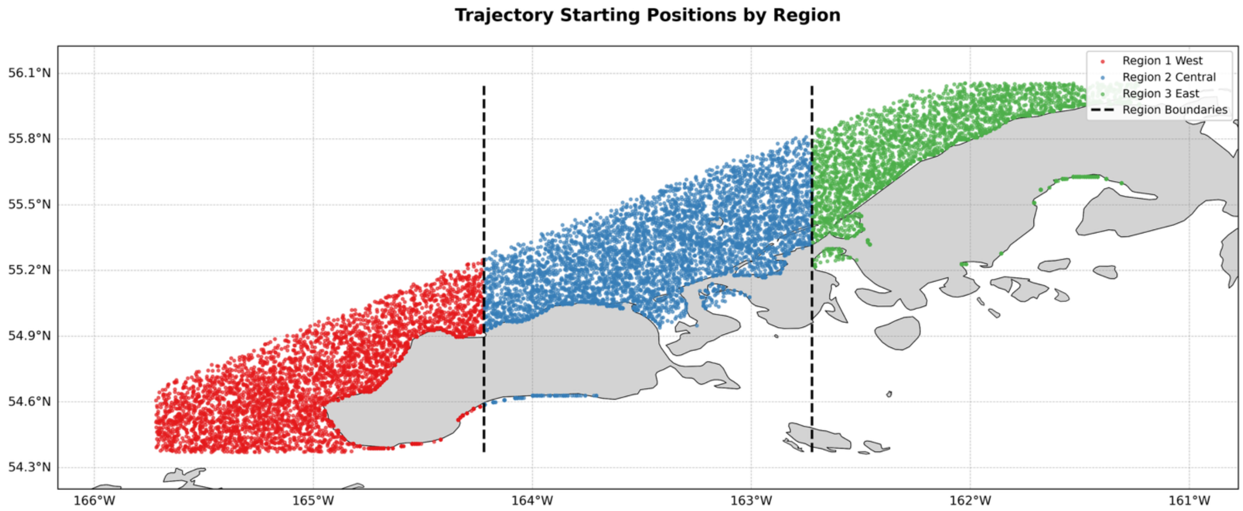

Supplementary Figure 5: Map showing the full domain where particles were released and the boundaries of each of the three regions along the coast of the Aleutians East Borough, Alaska, USA. Figure created with Python v3.11.9 using modules Cartopy v0.23.0, Seaborn v0.13.2, and Matplotlib v3.9.2.

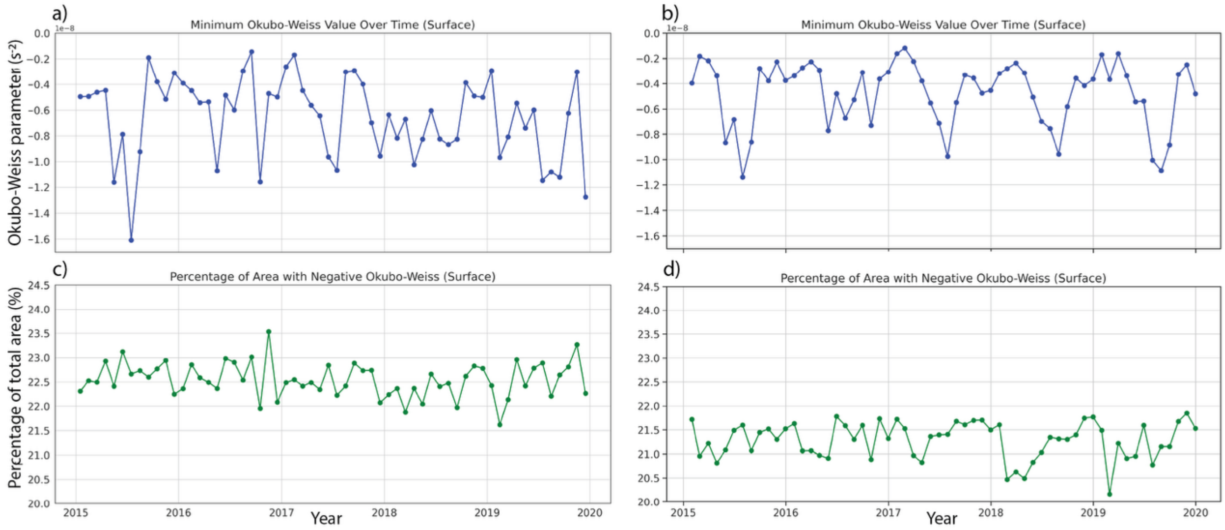

Supplementary Figure 6: Okubo-Weiss (OW) parameter calculated for the period 2015-2020 and area averaged. The upper panel shows the temporal variability of minimum OW parameter for a) GLORYS and b) SD-GLORYS. The percentage area with negative OW surface value is shown for c) GLORYS and d) SD-GLORYS. Figure created with Python v3.11.9 using modules Seaborn v0.13.2, and Matplotlib v3.9.2.

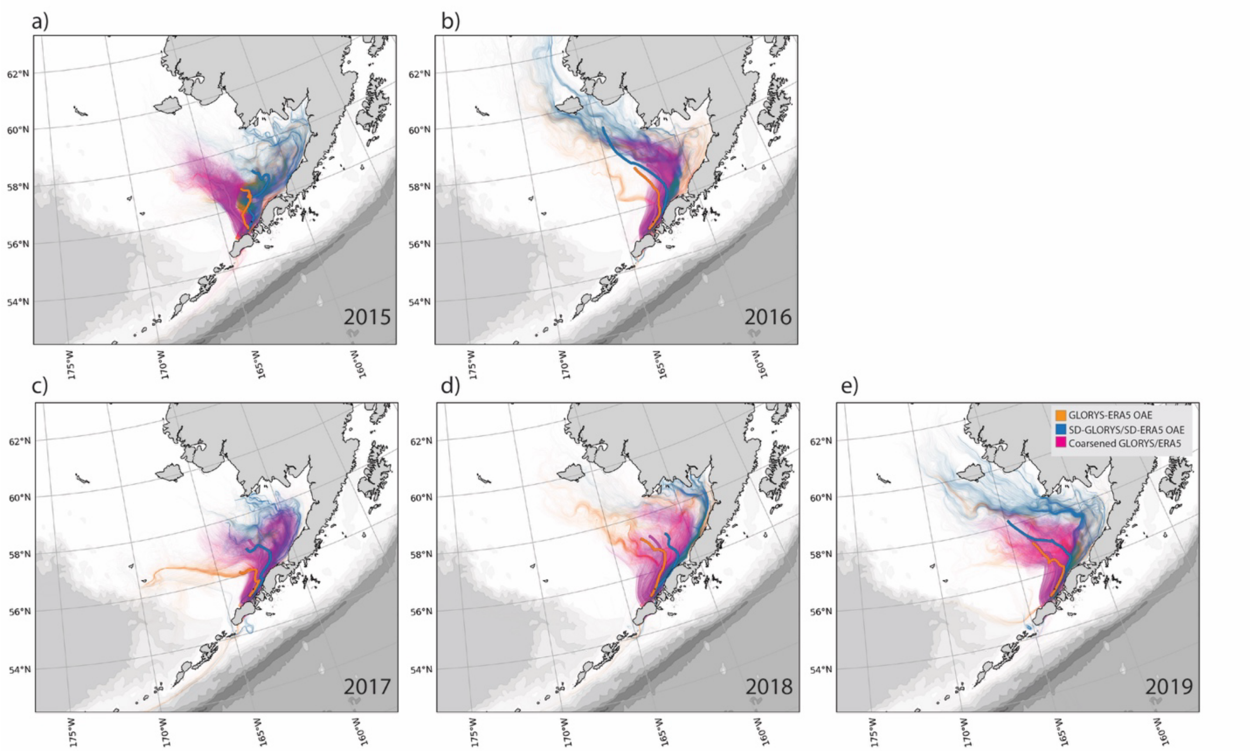

Supplementary Figure 7: Comparison between individual and mean particle trajectories tracked over 12 months (January through December) for the years 2015-2019 using ocean current fields from the high-resolution SD-GLORYS/SD-ERA5 downscaled flow fields (blue), the original GLORYS/ERA5 (orange) reanalysis, and coarsened GLORYS/ERA5 (fuchsia) for Central release sites along the coast of the Aleutians East Borough, Alaska. Figure created with Python v3.11.9 using modules Cartopy v0.23.0, Seaborn v0.13.2, and Matplotlib v3.9.2.

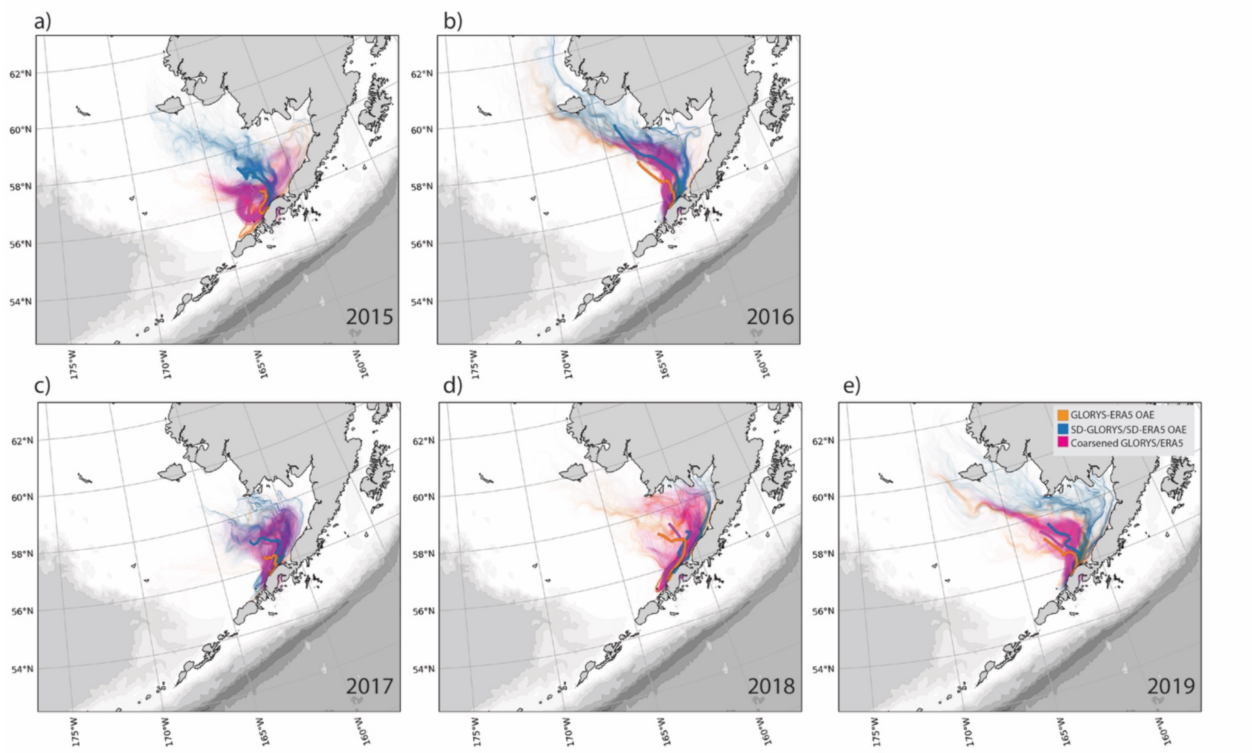

Supplementary Figure 8: Comparison between individual and mean particle trajectories tracked over 12 months (January through December) for the years 2015-2019 using ocean current fields from the high-resolution SD-GLORYS/SD-ERA5 downscaled flow fields (blue), the original GLORYS/ERA5 (orange) reanalysis, and coarsened GLORYS/ERA5 (fuchsia) for Eastern release sites along the coast of the Aleutians East Borough, Alaska. Figure created with Python v3.11.9 using modules Cartopy v0.23.0, Seaborn v0.13.2, and Matplotlib v3.9.2.
